# Emergency embolization of acute hemorrhage: cone-beam computed tomography with advanced planning and guidance software—a systematic review and case series

**DOI:** 10.1186/s42155-025-00639-7

**Published:** 2025-12-31

**Authors:** Ruben Geevarghese, Stephen B. Solomon, Francois H. Cornelis

**Affiliations:** 1 Division of Interventional Radiology, Department of Radiology, Memorial Sloan Kettering Cancer Center, New York, NY 10065 USA; 2 Weill Cornell Medical College, 1300 York Avenue, New York, NY 10065 USA

**Keywords:** Cone-beam computed tomography, Embolization, Therapeutic, Hemorrhage, Interventional radiology, Software

## Abstract

**Background:**

This study evaluates the current evidence on the use of cone-beam computed tomography (CBCT) combined with advanced planning and guidance (APG) software in transarterial embolization for acute hemorrhage and reviews our institution’s preliminary experience of use in emergency settings.

**Methods:**

A systematic review was conducted following the Preferred Reporting Items for Systematic Reviews and Meta-Analyses (PRISMA) guidelines. PubMed, SCOPUS, and Embase were searched to identify studies using CBCT with software for transarterial embolization of hemorrhage. Inclusion criteria focused on studies utilizing CBCT and APG software for hemorrhage management, with data extracted on demographics, hemorrhage locations, equipment/software specifications, technical success and procedural metrics. Additionally, we report a single-center review of patient outcomes using CBCT with multi-organ APG software (EmboASSIST with Virtual Injection, GE HealthCare, Chicago, IL, USA).

**Results:**

Nine studies met the inclusion criteria, including 71 patients. The most common site of bleeding was the lower gastrointestinal (GI) tract (62%). The mean technical success rate of embolization utilizing CBCT with APG was 94.3% (range: 82–100%). Three studies reported procedure time (mean 98.9 min, range 50–146 min), and two studies reported fluoroscopy time (mean 27.1 min, range 25–29.1 min). In our initial experience, all six cases were technically successful with favorable outcomes.

**Conclusions:**

CBCT with APG software is a feasible and effective tool for hemorrhage embolization in emergency settings. Its potential ability to improve bleeding detection compared to digital subtraction angiography (DSA) may lead to reduced procedure time, lower radiation exposure, and enhanced patient outcomes.

**Systematic review registration:**

NIHR-PROSPERO CRD 42024619227

## Background

Transarterial embolization is a key therapeutic option in managing acute hemorrhage [1], providing minimally invasive treatment with rapid hemostasis and low procedure-related morbidity. Although conventional computed tomography (CT) is a highly sensitive and non-invasive tool in the work-up of hemorrhage prior to intervention [2], digital subtraction angiography (DSA), performed during the procedure, can sometimes fail to detect bleeding identified on CT [2]. This may be related to a low flow rate of hemorrhage, an intermittent pattern of bleeding as well as overlapping vascular structures, resulting in inconclusive findings. Comprehensive interrogation by way of multiple selective DSAs may overcome this limitation and improve the detection rate with the trade-off of increasing procedure time, radiation dose, and contrast use. These limitations have spurred interest in advanced imaging modalities, particularly cone-beam computed tomography (CBCT) integrated with advanced embolization planning and guidance software [3], which has proven utility in liver, prostate, or head and neck embolization [3–9].

CBCT may offer enhanced intra-procedural hemorrhage detection and navigation capabilities. CBCT provides three-dimensional (3D) imaging of vascular and extra-vascular anatomy within the interventional suite [4]. CBCT-based advanced planning and guidance (APG) software can enable rapid, automated mapping of complex vasculature and identification of feeding vessels and optimal points of embolization with high accuracy, which are key factors for successful and timely embolization. While most APG software has been designed and approved for use in non-emergency liver embolizations, new multi-organ solutions such as Virtual Injection (Embo ASSIST with Virtual Injection, GE HealthCare, Chicago, IL, USA), which allows users to plan and guide embolizations in any anatomy, simulating embolization strategies from virtual injection points on automatically extracted 3D vasculature to identify feeding vessels, optimal embolization points, and determine the vascular path from a virtual catheter position. This functionality could be implemented in the emergency setting of hemorrhage embolization.

This study evaluates the current evidence for the use of CBCT combined with APG software in transarterial embolization for acute hemorrhage and reviews our institution’s preliminary experience in emergency settings.

## Methods

### Study selection criteria

A systematic review was undertaken on the use of CBCT with APG software in the transarterial treatment of hemorrhage and reported according to the Preferred Reporting Items for Systematic Review and Meta-Analysis (PRISMA) guidelines [10]. The review was registered in NIHR-PROSPERO (CRD 42024619227).

Inclusion criteria were (1) studies and case series treating patients with hemorrhage using transarterial techniques. (2) Use of CBCT in the treatment pathway to help in hemorrhage site identification. (3) Use of APG software to aid anatomy analysis, navigation, and/or transarterial treatment. Exclusion criteria were (1) conference abstracts. (2) Animal studies. (3) Articles published in a non-English language.

### Search strategy

The search strategy used in all instances was as follows: (artery OR vessel) AND embol* AND (hemor* OR haemor* OR bleed*) AND (cbct OR 'cb-ct' OR cone*). The search strategy was used in the PubMed, SCOPUS, and Embase databases.

### Study selection

A composite list of articles from the databases searched was evaluated, and duplicates were excluded. References in included articles were also analyzed as a source of further articles. Two reviewers (R.G and F.C) independently screened titles and abstracts using the Rayyan web application for systematic review screening [11], with the final articles for inclusion deemed eligible by consensus.

### Data extraction

Extracted data included study (publication year, study method, country of origin, study duration) and patient characteristics (number of patients, gender, age), site of hemorrhage, angiographic system, APG software, CBCT acquisition parameters (rotation, acquisition duration, and X-ray delay), contrast injection parameters (flow rate, delay, and duration), radiation dose, fluoroscopy time, procedure time, and technical success (defined as successful identification of the bleed on CBCT, segmentation of the arterial anatomy, and identification of the feeding vessels).

### Risk of bias assessment

The risk of bias was assessed using the Risk Of Bias In Non-randomized Studies of Interventions (ROBINS-I) tool [12] by two reviewers (R.G and F.C). Visual presentation of the results of the risk of bias assessment was created using the robvis application [13].

### Case series and APG software workflow

Retrospective analysis of the electronic medical record and picture archiving and communication system (PACS) was undertaken of emergency transarterial embolization procedures indicated for acute hemorrhage at a single institution between August 2021 and December 2024. Only cases wherein CBCT with APG software was used were included. Institutional review board approval was obtained (Memorial Sloan Kettering Cancer Center Institutional Review Board–Protocol Number: 16–402) and the need for informed consent was waived.

The institutional APG software workflow (Embo ASSIST with Virtual Injection, GE HealthCare, Chicago, IL, USA) comprises three steps. Firstly, automated segmentation of the opacified vessels on the CBCT is performed after selection of the artery from the origin of the vascular tree (typically the tip of the catheter used for contrast injection). Secondly, the user selects the target area/vessel of interest from the segmented vascular tree. The software then automatically and instantaneously delineates the path from the origin of the vascular tree to that point. Thirdly, the segmented vascular path from the origin of the vascular tree to the target embolization point can be automatically overlaid onto the live fluoroscopic images, allowing for a colored three-dimensional roadmap to aid prompt selective catheterization of the target vessels.

## Results

### Study selection and characteristics

**Fig. 1:**
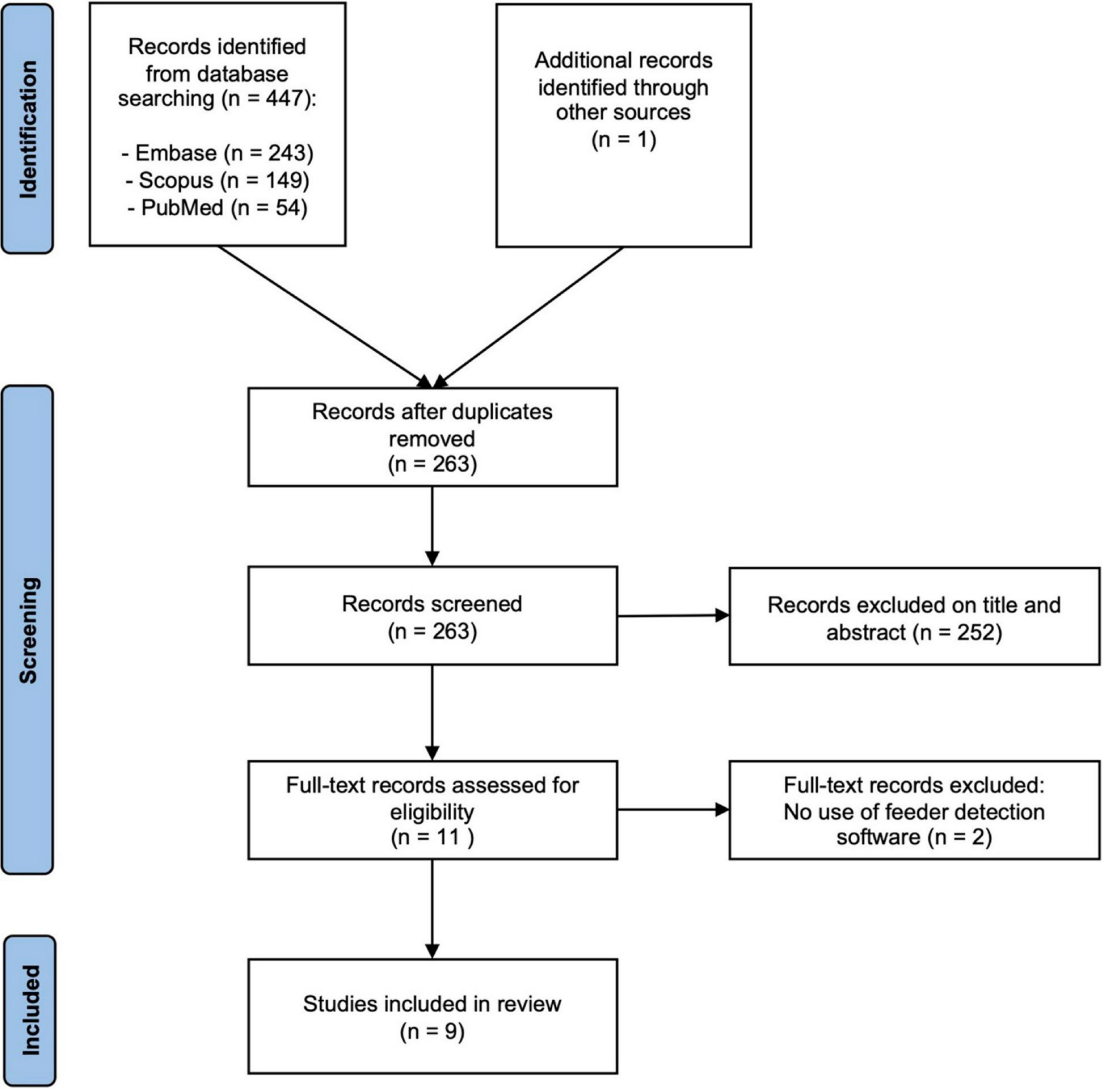
Study selection flowchart

A total of 448 articles were identified through electronic database searches and article reference review. Following the removal of duplicates, there were 263 articles remaining. Following title and abstract review, 252 articles were removed, with a total of 11 articles undergoing full text review. Of those undergoing full text review, 2 were excluded (due to the absence of APG software usage). A total of 9 studies were included in the final analysis after full text assessment. The study selection process is outlined in Fig. 1. The final 9 studies included a total of 71 patients who underwent transarterial treatment for hemorrhage using CBCT and APG software. The general characteristics and procedural data from the included studies are outlined in Tables 1 and 2, respectively.

**Table 1:** General characteristics of included studies

Study	Publication year	Methods	Country	Study duration	Number of patients	Age, mean (range)	Gender
Iwazawa et al. [14]	2014	Retrospective case series	Japan	2012–2013	5	52 (30–88)	3 male 2 females
Miyayama et al. [15]	2015	Retrospective case series	Japan	Not specified	2	56 (55–57)	1 male 1 female
Carrafiello et al. [16]	2016	Retrospective cohort study	Italy	2014–2015	20	62.4 (37–82)	10 male 10 females
Miyayama et al. [17]	2018	Retrospective case series	Japan	2016–2017	3	63.7 (60–68)	3 male
Sundararajan et al. [7]	2019	Retrospective case series	USA	2016–2018	2	66 (65–67)	1 male 1 female
Kamei et al. [18]	2021	Retrospective case series	Japan	Not specified	2	"70 s"^a^	2 male
Nakano et al. [19]	2022	Retrospective cohort study	Japan	2020	11	Not delineated	19 male 2 female
Pung et al. [20]	2023	Retrospective cohort study	USA	2020–2021	24	66.7 (not reported)	10 male 14 female
Hermie and Defreyne [21]	2023	Retrospective case series	Belgium	Not specified	2	57 (38–76)	2 female

^a^No further details were provided regarding age

**Table 2:** Summary of sites of bleeding, angiographic system, advanced planning and guidance (APG) software, cone beam CT (CBCT) parameters, contrast injection parameters, and technical success

Study	Site of bleeding (*n*)	Angiographic system	APG software	CBCT rotation (°)	CBCT acquisition time (s)	Imaging delay	Contrast injection rate (mL/s)	Contrast injection duration (s)	Fluoroscopy time, mean ± SD (min)	Procedure time, mean ± SD (min)	Radiation dose	Technical success
Iwazawa et al.	Pancreas (2), Jejunum, Colon, Bladder	Innova 3100, GE Healthcare	FlightPlan for Liver, GE HealthCare	200°	5	7–8	1–3	10	Not reported	100.4 ± 37.2	Not reported	100%
Miyayama et al.	Pancreas (2)	Not specified	EmboGuide, Philips Healthcare	240°	5.2	5	3	10	Not reported	Not reported	Not reported	100%
Carrafiello et al.	Kidney (5), Colon (5), Thigh (3), Jejunum (2), Duodenum (1), Spleen (1), Abdominal wall (1), Lung (1) and Pelvis (1)	Allura Xper FD20, Philips Healthcare	EmboGuide, Philips Healthcare	180°	8	3–15	1–20	Not possible to calculate	Not reported	50 ± 12.0	Not reported	90%
Miyayama et al.	Colon (2) and Pancreas (1)	AlluraClarity Xper FD20, Philips Healthcare	EmboGuide, Philips Healthcare	240°	5.2	5–30	3	Not reported	Not reported	Not reported	Not reported	100%
Sundararajan et al.	Pancreas (2)	Discovery IGS 740, GE Healthcare	Liver ASSIST, GE HealthCare	Not reported	Not reported	2–30	0.2–5	Not reported	Not reported	Not reported	Not reported	100%
Kamei et al.	Colon (2)	Azurion7 M20, Philips Healthcare	EmboGuide, Philips Healthcare	240°	5.2	7	0.2	15	Not reported	Not reported	Not reported	100%
Nakano et al.	Colon (9) and Other (2)	Azurion series 7 FD 20, Philips Healthcare	EmboGuide, Philips Healthcare	240°	5.2	2–6	0.6–1.2	Not reported	29.1 ± 13.9	146 ± 38.7	311 ± 207 mGy	82%
Pung et al.	Lower GI (24)	GE Discovery IGS7, GE Healthcare	Liver ASSIST, GE HealthCare	200°	5	5–22	0.5–5	22	25	Not reported	140.1 Gy.cm^2	100%
Hermie and Defreyne	Colon (1) and Jejunum (1)	Artis Q Angiography System; Siemens Healthineers	syngo Embolization Guidance, Siemens Healthineers	Not reported	Not reported	Not reported	Not reported	Not reported	Not reported	Not reported	Not reported	100%

### Risk of bias assessment

**Fig. 2:**
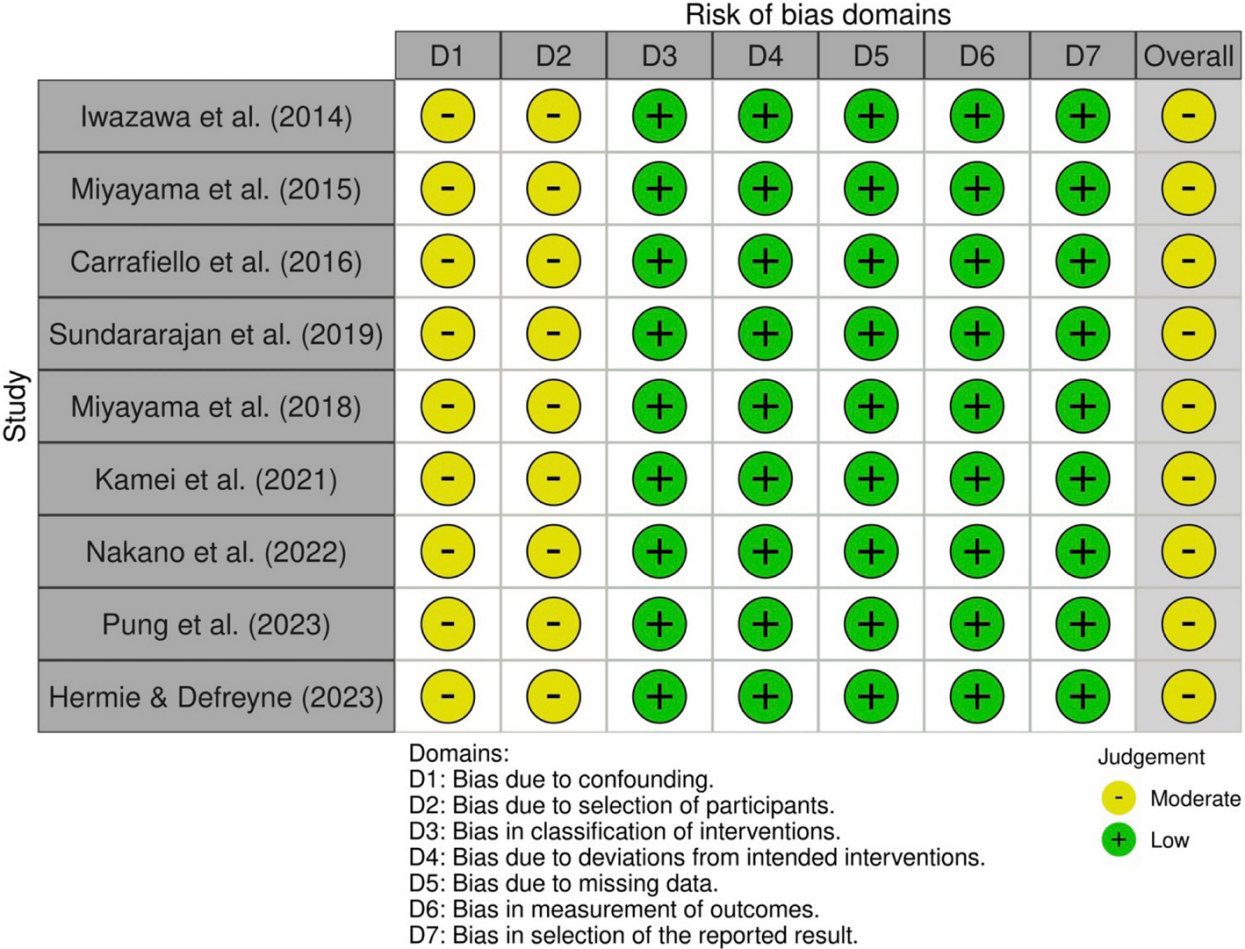
Risk of bias assessment using the ROBINS-I tool

The overall risk of bias for all nine included studies was moderate. All studies were classified as moderate risk of bias in the domains of confounding and bias in the selection of participants into the study. Complete risk of bias assessment (by domain and overall) is visually outlined in Fig. 2.

### Sites of hemorrhage and technical success

Sites of hemorrhage varied widely among studies, including colon/lower gastrointestinal (GI) tract (44), pancreas (7), kidney (5), jejunum (4), thigh (3), duodenum (1), spleen (1), bladder (1), lung (1), abdominal wall (1), pelvis (1), and other (2). CBCT with APG software was most commonly used in hemorrhage related to the lower gastrointestinal tract (62%, 44/71 patients). Technical success of embolization utilizing CBCT with APG software across all nine studies was 94.3% (67/71 patients).

### Angiographic systems and APG software

The angiographic systems and associated APG software used across studies varied widely and included models from GE HealthCare (Chicago, IL, USA), Philips Healthcare (Best, Netherlands), and Siemens Healthineers (Forchheim, Germany). The APG software used was the liver embolization planning and guidance solutions embedded in each angiographic system (Liver ASSIST (GE HealthCare, Chicago, IL, USA), EmboGuide (Philips Healthcare, Best, Netherlands), and syngo Embolization Guidance (Siemens Healthineers, Forchheim, Germany).

### Cone-beam computed tomography and contrast injection parameters

CBCT and contrast injection parameters demonstrated significant variation across studies. CBCT rotation angles ranged from 180° to 240°, and CBCT acquisition times were between 5 and 8 s. CBCT delay (following injection of contrast) varied substantially, with reported values from 2 to 30 s. Contrast injection rates ranged from 0.2 to 20 mL/s (median 2.7 mL/s), and injection durations ranged from 10 to 22 s (median 12.5 s).

### Procedure time, fluoroscopy time, and radiation dose

Three studies reported procedure time, the mean of which was 98.9 min (range 50–146 min). Two studies reported fluoroscopy time, the mean of which was 27.1 min (range 25 and 29.1 min). Finally, two studies reported radiation dose; however, this was expressed as dose area product in one study (140.1 Gy.cm^2^) and air kerma (311 mGy) in another study.

### Case series

Six patients were identified that met the inclusion criteria from the institution’s PACS. Two example cases are discussed below, with further details of all cases outlined in Table 3.

**Table 3:** Summary of institutional experience of advanced planning and guidance (APG) software with cone beam CT (CBCT)

Patient	Site of bleeding	Angiographic system	APG software	Fluoroscopy time, (min:seconds)	Procedure time, (min)	Air kerma, (mGy)	Kerma area product, (Gy.cm^2)	Technical success
1	Ileum	Innova 4100IQ, GE Healthcare	Embo ASSIST with Virtual Injection, GE HealthCare	16:43	111	808.9	140.1	Yes
2	Rectus sheath	Innova 4100IQ, GE Healthcare	Embo ASSIST with Virtual Injection, GE HealthCare	12:04	126	727.1	137.57	Yes
3	Retroperitoneum	Innova 4100IQ, GE Healthcare	Embo ASSIST with Virtual Injection, GE HealthCare	14:13	180	594	72.03	Yes
4	Ileum	Innova 4100IQ, GE Healthcare	Embo ASSIST with Virtual Injection, GE HealthCare	21:40	180	587.35	96.79	Yes
5	Cecum	Innova 4100IQ, GE Healthcare	Embo ASSIST with Virtual Injection, GE HealthCare	01:26:58	136	1052	175.41	Yes
6	Kidney	Allia IGS 7, GE Healthcare	Embo ASSIST with Virtual Injection, GE HealthCare	06:21	129	103.6	31.4	Yes

**Fig. 3:**
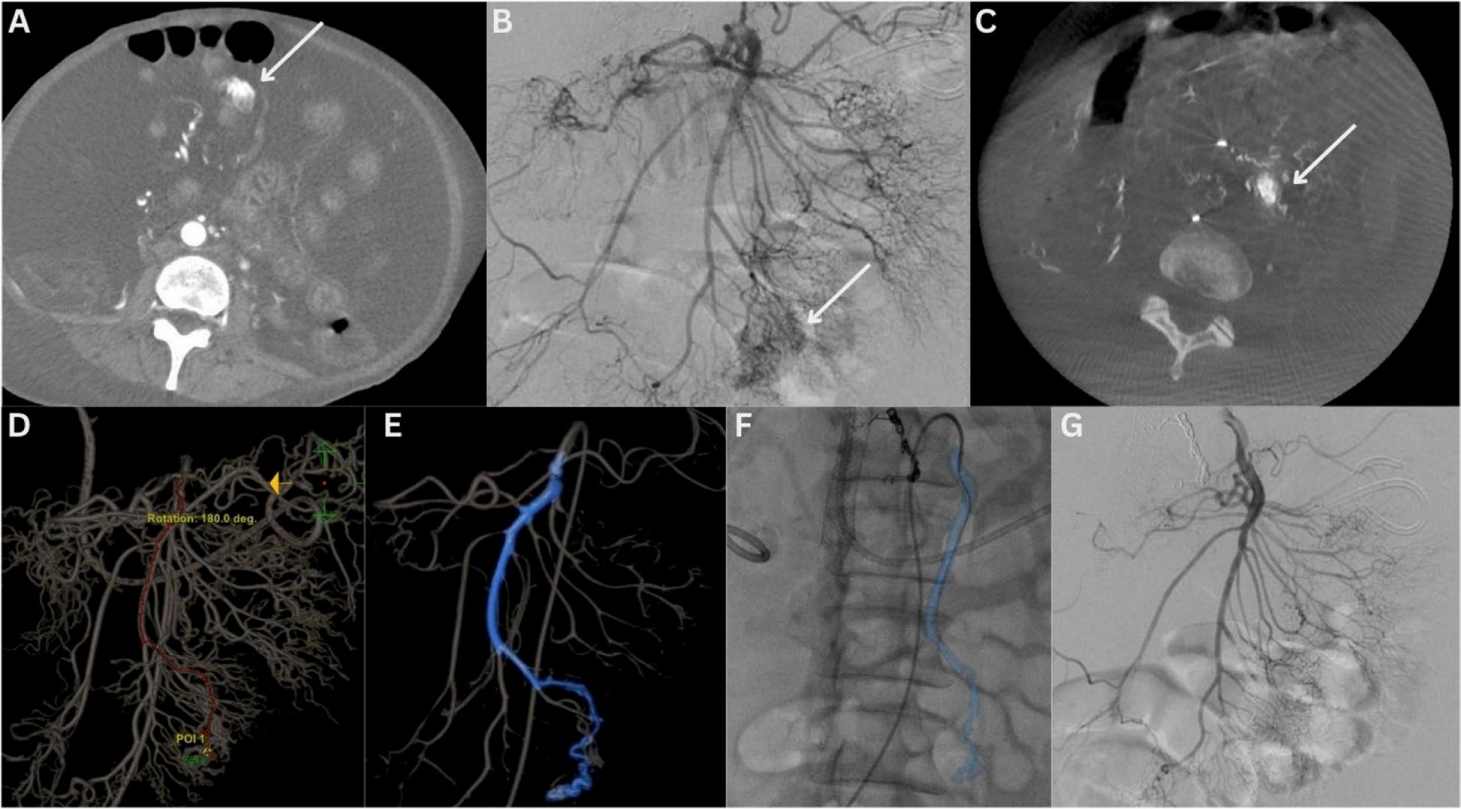
Case 1—Superior mesenteric artery embolization A 48-year-old woman presenting with lower gastrointestinal bleeding. **A** Focus of contrast extravasation (arrow) noted on the patient’s pre-procedural CT Angiogram. **B** No definitive focus of extravasation on a selective superior mesenteric artery (SMA) DSA. In retrospect an area of hypervascularity is noted in relation to an ileal branch (white arrow). **C** Given the patient’s large volume ascites and small bowel motion, the area of vascular abnormality (white arrow) now appeared to be positioned more centrally within the abdomen on the intra-procedural CBCT. **D** The arterial tree was automatically segmented and the point of interest was selected with automatic path calculation from the desired proximal position (Embo ASSIST with Virtual Injection, GE HealthCare, Chicago, IL, USA). **E** A colored three-dimensional roadmap is then generated from the processed data which can then be overlaid onto the live fluoroscopic image (**F**). **G** Post-glue embolization SMA DSA demonstrates successful post-embolization appearances

Patient 1 is a 48-year-old woman with a history of advanced pancreatic cancer treated with multiple courses of chemotherapy and radiotherapy, who was initially admitted with biliary sepsis. During the course of a prolonged admission, she developed melena and underwent CT imaging. A triple-phase CT angiogram suggested contrast extravasation in the small bowel on the arterial phase. She was transferred to the angiography suite (Innova IGS 540, GE HealthCare) and an emergency transarterial embolization was undertaken. Following catheter-directed selection of the superior mesenteric artery (SMA) and DSA acquisition, a focal vascular anomaly was seen arising from an ileal branch of the SMA. Given the patient’s ascites, overlap of bowel, and complexity of SMA branching, a CBCT (40°/s rotation speed, 200° rotation) was obtained from the origin of the SMA to aid in vessel navigation. Contrast injection parameters were 3 s x-ray delay, 5 mL/s for 8 s duration via a 5 French Cobra 2 catheter. The acquired CBCT was then used in the aforementioned APG software workflow to plan embolization. With the aid of the APG software, an ileal branch of the SMA was promptly selected using a 2.4 French Progreat microcatheter (Terumo; Somerset, NJ, USA), and 0.2 mL of n-butyl cyanoacrylate glue (TRUFILL n-BCA Liquid Embolic Agent, Johnson & Johnson Medtech, Warsaw, IN, USA, and Lipiodol Ultra Fluid [LUF], Guerbet, Aulnay-sous-Bois, France; dilution 1:4) was used to successfully embolize the area of suspected hemorrhage. A visual summary of the case is outlined in Fig. 3. Following embolization, the patient’s hemoglobin remained stable, but she had two further episodes of hematochezia, which resulted in a further CT angiogram. This demonstrated post-embolization appearances of the vascular anomaly with no new focus of contrast extravasation/hemorrhage. Her overall prognosis had worsened throughout the admission, and she was subsequently transferred to an inpatient hospice.

**Fig. 4:**
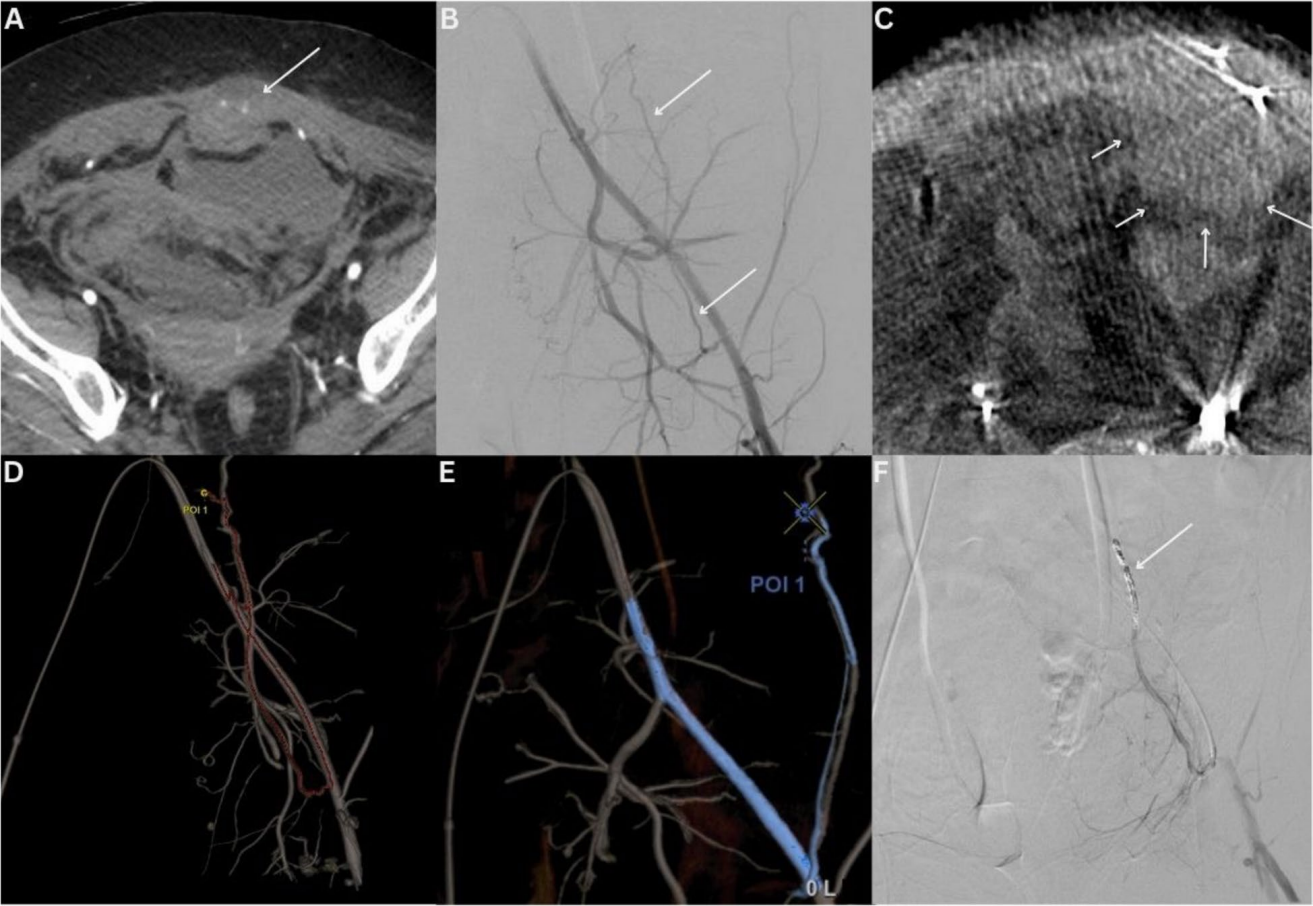
Case 2—Inferior epigastric artery embolization A 50-year-old woman presenting with bleeding into the left rectus abdominis. **A** Foci of contrast extravasation (arrow) noted on the patient’s pre-procedural CT Angiogram. **B** No definitive focus of extravasation seen from the left inferior epigastric artery (IEA–white arrows) on left iliac artery DSA. **C** Intra-procedural CBCT confirmed no evidence of contrast extravasation but an increase in size of the rectus sheath hematoma (white arrows). **D** The arterial tree was automatically segmented and the point of interest (correlating with the level of abnormal IEA noted on the positive pre-procedural CT) was selected with automatic path calculation from the desired proximal position (Embo ASSIST with Virtual Injection, GE HealthCare, Chicago, IL, USA). **E** A colored three-dimensional roadmap was then generated from the processed data. **F** Post-coil embolization left EIA DSA demonstrates successful post-embolization appearances

Patient 2 is a 50-year-old woman with a history of progressive glioblastoma multiforme who was initially admitted for agitation and altered mental status. During admission, she was diagnosed with a lower extremity deep venous thrombosis and pulmonary emboli for which she was started on anticoagulation. A few days later, she was admitted to the intensive care unit with shock which, on investigation, was diagnosed as hemorrhagic in etiology following the discovery of a large rectus abdominis hematoma, with accompanying foci of arterial phase contrast extravasation on a triple-phase CT angiogram. She underwent transfusion with 2 units of blood and was transferred to the angiography suite for emergency embolization (Innova IGS 540, GE HealthCare). Following catheter-directed (5 French Berenstein) selection and DSA acquisition of the left common iliac and left inferior epigastric (IEA) arteries, no contrast extravasation was seen in the left rectus abdominis. Given the negative DSA, a CBCT (40°/s rotation speed, 200° rotation) was obtained from the origin of the left IEA, using a 2.4 French Progreat microcatheter (Terumo; Somerset, NJ, USA). Contrast injection parameters were 3 s X-ray delay, 2 mL/s for 8 s duration. No foci of extravasation were seen on the initial review of the acquired CBCT. The aforementioned APG workflow was used to segment and evaluate the vascular tree, evaluate and confirm the absence of contrast extravasation, and utilize the segmented data for augmented fluoroscopy to conduct the embolization in the target segment of the left IEA. Microcoils (2 × 2 mm and 2 × 3 mm) were used to empirically treat the left IEA. A visual summary of the case is outlined in Fig. 4. Following embolization, the patient’s hemoglobin remained stable. Despite the embolization, the patient’s impaired mental status continued to deteriorate. She was subsequently transferred to an inpatient hospice 2 days after the embolization.

## Discussion

This review demonstrates that CBCT with advanced planning and guidance (APG) software has demonstrated effectiveness in transarterial embolization for acute hemorrhage management, offering advantages in emergency settings. Existing literature highlights the successful application of CBCT with APG software across diverse vascular territories, employing varying imaging and contrast protocols. Our preliminary institutional experience supports these findings, with CBCT and APG (Embo ASSIST with Virtual Injection, GE HealthCare, Chicago, IL, USA) facilitating multi-organ embolizations and aiding in the successful management of acute hemorrhage.

Conventional catheter-directed angiography relies on two-dimensional DSA, which can be limited in detecting subtle or intermittent bleeding, particularly in non-selective acquisitions. CBCT, by providing three-dimensional imaging within the interventional suite, addresses these limitations and enables a more comprehensive assessment of vascular anatomy. Studies have shown that CBCT with APG can identify bleeding sources undetected by DSA. For instance, Pung et al. reported that in 46% of lower GI bleed cases with inconclusive DSA, CBCT with APG identified bleeding in 42% of cases, leading to changes in intraprocedural management. Similarly, Carrafiello et al. demonstrated that CBCT with APG improved bleeding site detection in 95% of cases with inconclusive DSA. The role of CBCT with APG could therefore be prioritized early in contexts such as where there is negative/inconclusive DSA in patients with a previously positive CT evidence of bleeding and/or in patients who are hemodynamically unstable at the time of embolization. Following the outcome of CBCT, further strategies for management of bleeding, such as provocative angiography [14] or organ/territory permitting, empiric embolization [15], could be employed.

CBCT with APG software offers several workflow enhancements, including fewer image acquisitions, lower radiation dose, shorter fluoroscopy time, and potentially decreased contrast volume usage. These improvements stem from the software’s ability to provide detailed imaging at the outset, aiding in the identification of feeding vessels and optimal embolization points. Additionally, a live 3D roadmap functionality enhances fluoroscopy guidance, expediting navigation to target vessels. Of note, advances in fluoroscopic technology have also demonstrated reductions in radiation dose during hemorrhage embolization [16]. Overall, routine implementation of CBCT with APG could therefore help to standardize workflows in high-stakes hemorrhage management.

Despite its benefits, CBCT with APG software presents challenges. Acquisition and analysis can be time-intensive, particularly in emergency settings. Importantly, our institutional workflow allowed for accurate extraction and guidance to be achieved in under five minutes. Furthermore, the time required for CBCT and APG use is likely to be offset by reductions in overall procedure time. Standardizing its use in routine cases could further improve efficiency in emergency scenarios.

The impact of APG software on interventional radiology (IR) training is another consideration. While APG facilitates navigation, it may limit the development of certain technical skills. While APG software facilitates procedural guidance, it may potentially impact the development of traditional two-dimensional fluoroscopic angiography interpretation skills, particularly in trainees. Operators must still physically navigate catheters to target vessels; however, the reliance on software-generated roadmaps may reduce exposure to conventional angiographic interpretation. Additionally, CBCT interpretation requires specific training, as the imaging appearances can differ significantly from pre-procedural CT angiography despite their similarities. Further research is needed to evaluate the long-term effects of APG on skill acquisition.

The studies reviewed revealed significant variability in CBCT and contrast injection parameters. Detailed justification for specific CBCT acquisition and contrast injection protocols was not provided. The variability likely reflects institutional preferences, available equipment capabilities and operator experience. Future standardization efforts would benefit from prospective studies comparing different protocol parameters to establish evidence-based guidelines. Many studies utilized APG software initially designed for liver embolization, requiring workflow adjustments for non-hepatic applications. Our preliminary experience with multi-organ APG software demonstrated improved automatic segmentation and vessel identification, streamlining the workflow. Future studies should incorporate advanced, organ-agnostic APG software, such as EmboASSIST with Virtual Injection (GE HealthCare, Chicago, IL, USA), to optimize imaging quality and procedural performance.

The systematic review revealed several limitations, including small sample sizes, retrospective designs, and moderate risk of bias due to selection and confounding factors. The absence of comparator arms further limits definitive conclusions. Additionally, the reporting of key metrics such as procedure time, fluoroscopy time, and radiation dose was inconsistent. Standardized protocols for CBCT acquisition and radiation dose reporting are essential for reproducibility and comparability. Future research should focus on larger, prospective studies with standardized imaging workflows and comparator arms to evaluate the true utility of CBCT with APG in hemorrhage management. The integration of artificial intelligence-driven software could further enhance procedural efficiency and outcomes, building a robust evidence base for this promising technology.

Despite the benefits and widespread availability of CBCT-capable angiography systems, several factors may explain the limited routine integration into emergency embolization of acute hemorrhage workflows. First, the perception of additional time of acquisition and analysis may be deemed prohibitive by some operators (despite being potentially offset by procedure time as the intervention proceeds and concludes). Second, a proportion of interventional radiologists and supporting team (e.g., radiation technologists) may lack familiarity with the nuances of the technology and its optimal application in hemorrhage scenarios. Third, institutional protocols and workflows are often established based on conventional angiography, and changing ingrained practice patterns requires dedicated effort and training. Fourth, reimbursement structures may not adequately recognize the additional time and expertise required for CBCT with APG procedures. The above factors are also likely to influence the paucity of larger and more contemporary studies.

In conclusion, CBCT with APG software is a valuable tool to assist the performance of hemorrhage embolization, particularly in emergency settings. Its ability to enhance bleeding detection, streamline workflows, and reduce procedure metrics underscores its potential to improve patient outcomes. Continued advancements in software and standardized protocols will further refine its role in IR practice.

## Data Availability

Data is available from the authors upon reasonable request.

## Abbreviations

APG: Advanced Planning and Guidance
CBCT: Cone-beam computed tomography
GI: Gastrointestinal
IR: Interventional radiology
PACS: Picture Archiving and Communication System
PRISMA: Preferred Reporting Items for Systematic Reviews and Meta-Analyses
ROBINS-I: Risk Of Bias In Non-randomized Studies of Interventions
SMA: Superior mesenteric artery
